# Haff disease: overview and clinical features

**DOI:** 10.1590/S1678-9946202466052

**Published:** 2024-09-06

**Authors:** Gabriel Rotsen Fortes Aguiar, Roberto Cesar de Moura Silva, Karla Cristina Silva Petruccelli, Michael Nascimento Oliveira, Gabriel Antônio Uchôa de Brito, Polianna Lemos Moura Moreira Albuquerque, Elizabeth De Francesco Daher, Geraldo Bezerra da Silva

**Affiliations:** 1 Universidade Federal do Ceará, Faculdade de Medicina, Departamento de Medicina Clínica, Programa de Pós-Graduação em Ciências Médicas, Fortaleza, Ceará, Brazil; 2 Universidade de Fortaleza, Programa de Pós-Graduação em Ciências Médicas, Fortaleza, Ceará, Brazil; 3 Universidade Federal do Amazonas, Faculdade de Medicina, Manaus, Amazonas, Brazil; 4 Hospital José Mendes, Itacoatiara, Amazonas, Brazil

**Keywords:** Haff disease, Haff syndrome, Rhabdomyolysis, Amazon, Epidemiology

## Abstract

Haff disease was first described at the beginning of the twentieth century in Europe. Almost a century later, thousands of cases have now been reported in different countries. In Brazil, most cases are observed in the Amazon region, and its associated factors remain to be fully understood. This disease is an uncommon syndrome characterized by intense myalgia and rhabdomyolysis, which manifests within 24 h after consuming some types of freshwater or saltwater fish and crustaceans. A possible heat-stable toxin contained in seafood may be the cause of Haff disease, but this hypothesis is not yet completely proven. This review will describe the clinical and epidemiological aspects of Haff disease with updated literature.

## INTRODUCTION

Haff disease is an uncommon syndrome characterized by intense myalgia and rhabdomyolysis, which manifests within 24 h after consuming some types of freshwater or saltwater fish and crustaceans. The implicated cause is believed to be a heat-stable toxin. The term “Haff”, rooted in the German language, signifies a shallow lagoon, potentially hinting at this condition’s etiological agent. The expanding global seafood trade and consumption, coupled with the burgeoning growth in international tourism, are anticipated to contribute to a heightened incidence of Haff disease^1-4^. Particularly noteworthy is the increasing prevalence of Haff disease in the Amazon region, where the factors behind this rise remain enigmatic and need further investigation.

### History and epidemiology

In 1924, the initial instances of Haff disease were documented in the vicinity of the Haff coasts, situated along the Baltic Coasts of Russia and Poland. Later, more than 1,000 cases were reported in the subsequent years. Notably, the disease was characterized by a seasonal pattern, predominantly occurring during the summer and autumn months in this particular region^2,5^. Since its initial observed occurrence, numerous countries across the globe have consistently reported the recurrence of Haff disease in distinct seasonal clusters^2,3,6,7^.

As of the present moment, the United States of America (USA) and China have reported the largest number of cases. Nevertheless, outbreaks have been documented in many other countries, such as Brazil, particularly in the vicinity of the Amazon River. Over the past few years, there has been a gradual increase in global case clusters^1,5,8^.

While isolated cases and small outbreaks are more prevalent, larger occurrences have been documented. One notable outbreak was recorded in China, where hundreds of cases were reported^8^. During an outbreak in Brazil, a significant attack rate of 55% was identified among individuals who shared a fish meal^7^. Studies conducted in China unveiled a discernible trend, indicating that outbreaks predominantly occur during summer, primarily impacting adults. This observation could be associated with lesser popularity of seafood consumption among children in that demographic^1^.

### Etiology and epidemiology

The etiology of Haff disease is believed to be linked to a heat-stable toxin present in seafood. However, efforts to isolate and characterize this toxin have been unsuccessful. Palytoxin, identified in marine fish, has been correlated with instances of rhabdomyolysis and could potentially serve as a model for conducting further investigations into the suspected toxin responsible for rhabdomyolysis following the consumption of freshwater fish^1,9^.

Across the timeline of Haff disease outbreaks, multiple causative agents have been identified. Table 1 presents a compilation of Haff disease cases across different countries. This compilation aims to assess the diversity of causative agents involved and illustrate variations in incidences regarding age, sex, and other factors.

A case series from 2000 to 2016 in China revealed that crayfish was implicated in most of the Haff disease outbreaks in the country. Notably, there were two exceptions, in which pomfret (*Colossoma brachypomum*) and “lobsters” from Dongting Lake were identified as the responsible agents^1^.

In 2008, a noteworthy outbreak of Haff disease comprising 27 reported cases occurred in the Amazon region of Brazil. The individuals affected had consumed pacus (*Mylossoma spp.*), tambaqui (*Colossoma macropomum*), or Red-Bellied Pacu (*Piaractus brachypomus*)^10^.

**Figure 1:**
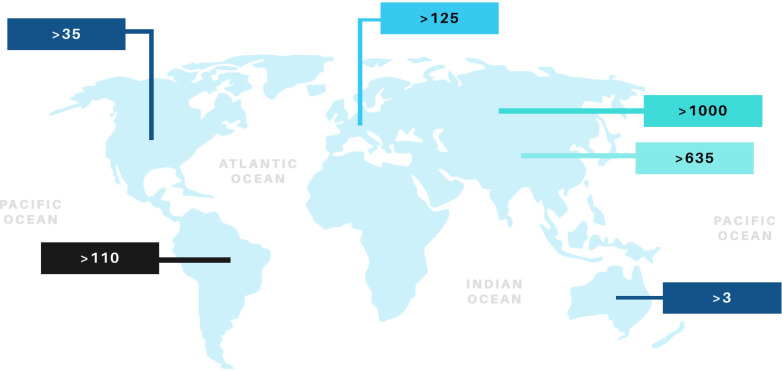
Haff disease cases worldwide^4^,^7^,^12^-^20^.

Outbreaks in the USA have been linked to the consumption of buffalofish (*Ictiobus spp.*), crawfish, or salmon^11^. Figure 1 illustrate the distribution of Haff disease cases worldwide^12-20^.

According to the Amazonas Health Surveillance Foundation^21^, recent cases of Haff disease have been documented in the region, specifically during the years 2022 and 2023, with most cases concentrated in the Amazonas State. In 2022, there were a total of 103 confirmed cases, followed by 61 cases reported between January and October 2023. Out of these 61 cases reported in 2023, 62% of the infections occurred in urban areas, while 38% were in rural areas. Additionally, associated comorbidities were outlined: 57% of the patients had hypertension, 50% had diabetes, and 14% had asthma. The most prevalent age group among these individuals was between 40 and 59 years old. The species associated with rhabdomyolysis in these cases were the pacu (*Mylossoma spp.*) and tambaqui (*Colossoma macropomum*)^21^. However, specific data concerning clinical history and surveillance mechanisms for this population have not been published.

### Clinical presentation

Haff disease is characterized by unexplained myalgia, often accompanied by rhabdomyolysis that occurs within 24 h after consuming specific types of cooked freshwater fish or crustaceans. The pathophysiological mechanisms underlying this condition are not yet fully comprehended. Myalgia associated with Haff disease can manifest as severe, moderate, or mild discomfort, affecting muscles in various areas such as the back, shoulders, neck, chest, upper and lower limbs. The intensity of muscle pain correlates directly with the estimated weight of the consumed fish. Nevertheless, clinical presentation may show symptoms indicative of multi-organ involvement. Individual susceptibility varies, and some individuals may remain asymptomatic even after sharing the same seafood meal as those affected^1,16^.

### Diagnosis

The diagnosis of Haff disease relies on the patient’s history, and the symptoms can show variations. Typically, a clinical presentation featuring unexplained myalgia and rhabdomyolysis, accompanied by a significantly elevated creatine kinase (CK) as fivefold or more above the normal level, along with a positive history of consuming cooked seafood within the past 24 h, is indicative of the condition. Special attention should be given to the history-taking process, as, even though the disease is colloquially known as “black urine disease” in many countries, this specific symptom is observed in only about 40% of cases. Mild presentations, encompassing general and gastrointestinal symptoms, may also occur, potentially leading to a delayed or overlooked diagnosis^1^. The absence of an exclusive biomarker or a distinctive pattern among existing biomarkers could contribute to diagnostic challenges, emphasizing the need for researchers to address this issue thoroughly.

### Gastrointestinal and hepatic involvement

Gastrointestinal symptoms were outlined in a literature review from China^1^. A man was the subject of a case report, wherein he was admitted with manifestations of gastroenteritis, encompassing symptoms such as nausea, vomiting, diarrhea, and myalgias, after attending to a seafood buffet. Laboratory tests revealed elevated levels of aspartate transaminase (AST) and alanine transaminase (ALT) (174 and 63 IU/L, respectively)^11^. Laboratory abnormalities are primarily characterized by increased serum levels of myoglobin and creatine kinase (CK). However, other enzymes such as lactate dehydrogenase (LDH), AST, and ALT may also experience elevation. The reddish-brown appearance of the urine is attributed to myoglobinuria^1,8^.

In another study from China, an analysis of 495 cases of Haff disease revealed average levels of AST at 164.6 ± 207 IU/L and ALT at 85.9 ± 88.2 IU/L^3^. Upon admission to the hospital, 13.2% (9 out of 68) of patients diagnosed with Haff disease presented nausea or vomiting. The study also detailed hepatic enzyme levels, indicating that in a subgroup of 46 patients, AST levels upon admission were recorded at 292.8 ± 74.7 IU/L, while ALT levels were measured at 184.9 ± 61.3 IU/L^17^.

Abdominal pain, dry mouth, dizziness, hypersensitivity to touch, muscle contractions, general discomfort, diarrhea, chest tightness, headaches, increased blood pressure, and the presence of brown or tea-colored urine are additional symptoms documented in outbreaks reported in China, USA, and Brazil. These symptoms typically manifest within the initial hours following seafood consumption^1,2,6,14,18^.

### Kidney involvement

Haff disease can complicate with kidney involvement, given that rhabdomyolysis is a significant aspect of this syndrome. Myoglobin, discharged from muscle breakdown, can potentially obstruct renal tubules after filtration by the glomerulus, leading to toxicity. In individuals with Haff disease, standard urinalysis may also detect the presence of blood or protein in the urine^4,8^. In a retrospective cohort study conducted in a Chinese hospital, 68 patients diagnosed with Haff disease were reported, and among them, 7 individuals had hematuria at the time of their initial presentation^16^. That hematuria can, in fact, be myoglobinuria, as dipstick cross-react with myoglobin and appear as hematuria^19^. In a China-based outbreak involving 29 patients, hematuria was observed in 9 instances, and proteinuria was identified in 13 cases^20^.

Research conducted in Brazil and Australia has documented the presence of acute kidney injury (AKI) in 13% and 33% of cases, respectively^10,22^. In a case report from Poland, a 38-year-old man with pre-existing arterial hypertension experienced the development of AKI during his course of illness. This was marked by an elevation in creatinine levels to 6.11 mg/dl, along with the manifestation of dark-colored urine, proteinuria, and hematuria^14^. In a retrospective study examining 16 cases of Haff disease in China, hematuria was observed in 50% of cases, while 31% had proteinuria. All patients experienced liver dysfunction, and one developed AKI^23^.

A 2016 outbreak in China evidenced abnormal levels of urea and creatinine, recorded in 32.4% and 44.1% of cases, respectively. Further analysis of a subgroup consisting of 50 individuals revealed proteinuria in 18% and hematuria in 20% of cases^3^.

In another retrospective cohort study in China, blood urea nitrogen (BUN) at admission was 7.3 ± 2.1 mmol/L. In another Chinese outbreak, 7.4% of cases showed slightly elevated BUN, while 3.7% had abnormal creatinine levels^15,16^.

While severe kidney dysfunction is rare due to effective volume repletion and other rhabdomyolysis management strategies preventing kidney injury, renal failure can still occur, albeit rarely^1,23^. Consequently, some patients may need renal replacement therapy. As exemplified in the previously mentioned Polish case, renal replacement therapy became imperative during hospitalization^14^.

### Laboratory findings

Haff disease typically manifests with elevated creatine kinase (CK) levels. In an examination of 46 patients admitted to a Chinese hospital with Haff disease, CK and CK-MB reached levels of 5,771.2 ± 2,645.5 IU/L and 535.5 ± 162.1 IU/L, respectively. Another outbreak in China revealed a median CK level of 1,302 IU/L^15^. Lactate dehydrogenase (LDH) also increased, reaching levels of 739.7 ± 261.4 IU/L^17^.

**Figure 2:**
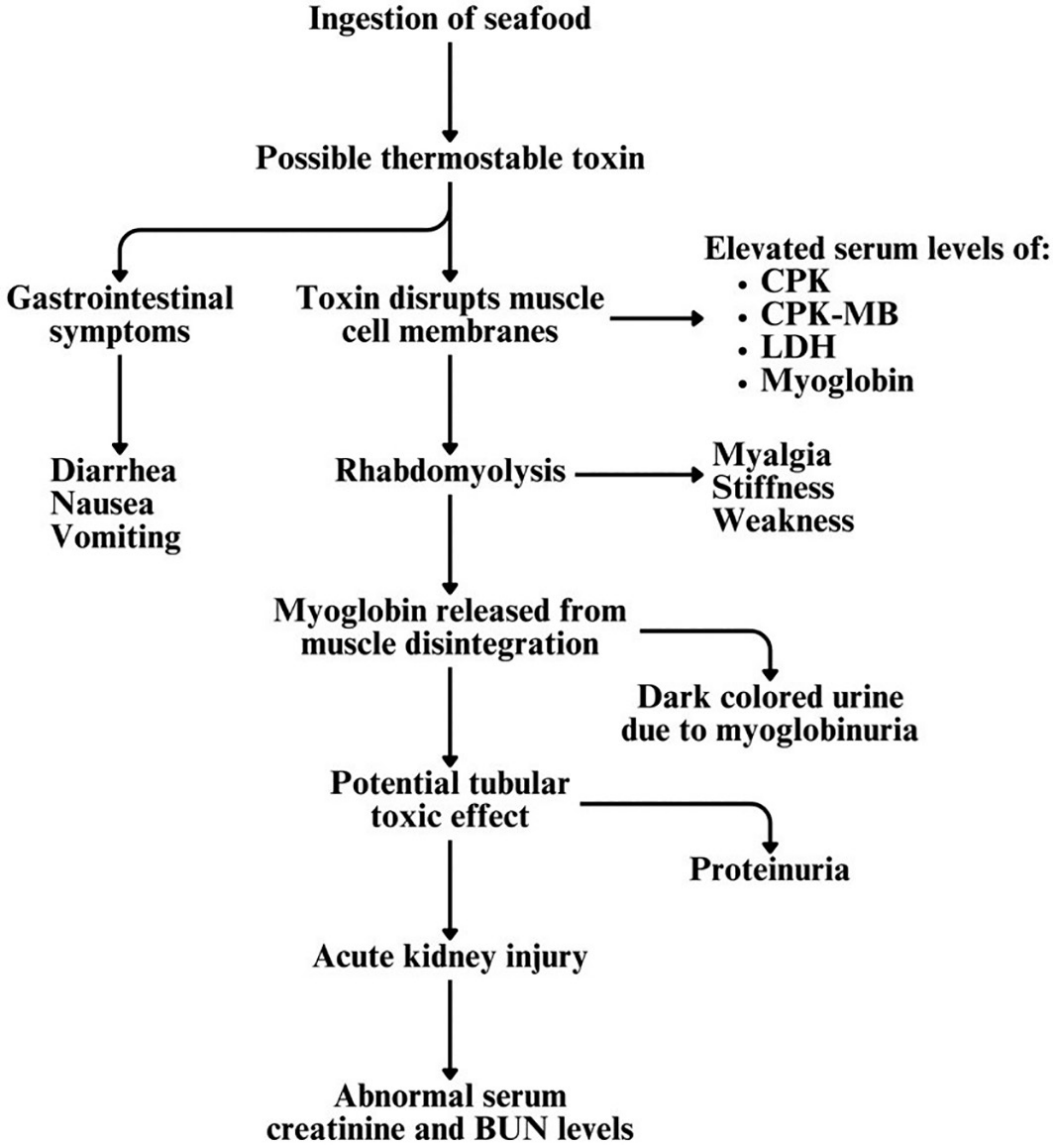
Pathophysiology of Haff disease.

A cohort study involving 495 Haff disease patients in China indicated altered myoglobin levels, with a mean value of 330.0 ± 121.2 ng/mL and CK levels at 5,439.2 ± 4,765.1 IU/L^3^. In a separate 2016 outbreak in China, median CK levels were reported at 2,445.0 U/L, median CK-MB at 110.0 U/L (72.5 to 155.0), and myoglobin was detected in 96.5% of cases, with median values of 1,020.0 IU/L (237.6 to 6,397.3)^20^. The pathophysiology of Haff disease is illustrated in Figure 2.

Prognosis

In general, prognosis of Haff disease is good, with most individuals experiencing full recovery within a few days. Nonetheless, severe complications may arise, including death due to multi-organ failure and prolonged myopathy^1^.

A case report from China illustrated a fatal outcome of a 66-year-old man with a 20-year history of hypertension. The individual developed diffuse myalgia, oliguria, coffee-colored urine, shortness of breath, tachycardia, tachypnea, hypotension, and died due to multiple organ failure approximately two days after consuming crayfish^24^.

### Treatment and recovery

Fluid therapy, urine alkalinization, and other supportive measures, such as kidney and liver function preservation and electrolyte monitoring are essential^23^.

Mild presentations are most common, with almost all affected subjects fully recovering after no more than one week with supportive treatment. Chronic muscle injury has already been described, but generally myalgia and muscle weakness should normally subside within 2 to 3 days, and serum CK become normal within 5 to 6 days^1,5,15^. In a study with 16 cases from China, myalgia disappeared in all patients at 24 to 72 h after treatment, and liver function and CK level gradually returned to normal after 72 h^23^.

## CONCLUSION

Haff disease seems to have a worldwide distribution, as observed in reports from last decades. For any subject presenting a sudden onset of myalgia, weakness, and other symptoms of rhabdomyolysis, seafood consumption should be investigated in the history. Haff disease must be included in differential diagnoses of rhabdomyolysis. Patients can show gastrointestinal symptoms in initial presentation. Kidney function tests can be abnormal, and urinary findings, such as proteinuria and hematuria, can occur. Prognosis is good, but complications such as AKI, chronic myopathy, and multi-organ failure can also occur. Further research is needed to characterize the responsible toxin, attack rates, and preventive measures. The Amazon region can be considered a vulnerable area for Haff disease, and further studies should be conducted to better elucidate its cause and associated factors.
